# Development of a rain down technique to artificially infest hemlocks with the hemlock woolly adelgid, Adelges tsugae

**DOI:** 10.1093/jis/14.1.106

**Published:** 2014-08-01

**Authors:** Robert M. Jetton, Albert E. Mayfield, Zaidee L. Powers

**Affiliations:** 1 Research Assistant Professor, Camcore, Department of Forestry &Environmental Resources, North Carolina State University, Raleigh; 2 Research Entomologist, USDA Forest Service, Southern Research Station, Asheville, NC; 3 Graduate Research Assistant, Camcore Department of Forestry &Environmental Resources, North Carolina State University, Raleigh

**Keywords:** exotic species, host resistance screening, artificial infestation, *Tsuga canadensis*, *Tsuga canadensis*

## Abstract

The hemlock woolly adelgid *Adelges tsugae* Annand (Hemiptera: Adelgidae), is a non-native invasive pest that has caused widespread decline and mortality of eastern hemlock ( *Tsuga canadensis* (L.) Carr. (Pinales: Pinaceae)) and Carolina hemlock ( *T. caroliniana* Engelm.) in the eastern United States. Our preliminary experiments evaluated the utility of a rain-down technique to induce artificial infestations of *A. tsugae* on hemlock seedlings en masse. Experiments were conducted in PVC (1 m ^3^ ) cages topped with poultry wire for placement of *A. tsugae* -infested branches, and with 1 m ^2^ gridded glue sheets and/or hemlock seedlings placed below to capture adelgid abundance, distribution, and infestation rate data. In the March 2011 experiment, the density of progrediens crawlers (adelgid nymphs, first instars) that rained down inside the PVC cages was significantly higher in the high ovisac treatment compared to the low ovisac treatment, with an estimated 513,000 and 289,000 crawlers per m ^2^ falling beneath each treatment, respectively. Resulting *A. tsugae* infestation rates on Carolina hemlock seedlings placed inside the cages did not differ between the treatments but were at or above established damage threshold densities for the adelgid. Infestation rates on eastern hemlock seedlings that were placed in cages nine days after the experiment started were below damage threshold levels and did not differ between the treatments. In the May 2011 experiment, the density of sistens crawlers raining down was substantially lower, with 17,000 and 33,000 falling per m ^2^ in the low and high ovisac treatments, respectively. Resulting infestation rates on Carolina hemlock seedlings were extremely low and well below damage threshold levels. Although *A. tsugae* crawlers were well distributed across the 1 m ^2^ gridded glue sheets placed at the bottom of each cage, hot spots of unusually high crawler density did occur in both experiments. This rain-down technique shows potential for use in an operational tree-breeding program where screening large numbers of hemlock seedlings for resistance to *A. tsugae* is required.

## Introduction

The hemlock woolly adelgid, *Adelges tsugae* Annand (Hemiptera: Adelgidae), is a serious invasive forest pest in the United States, where it poses a significant threat to the long-term sustainability of hemlock *(Tsuga* spp. (Pinales: Pinaceae)) ecosystems. The insect was first described from specimens collected in western North America, where it was initially thought to be an exotic pest of western hemlock *(T. heterophylla* (Raf) Sarg.) ( Annand 1924 ) but is now known to be native ( Havill et al. 2011 ). The adelgid is exotic in eastern North America, where it was introduced into Virginia in the early 1950s ( Havill et al. 2006 ), most likely on ornamental nursery stock imported from Japan. The distribution of *A. tsugae* in this region now covers a 19-state area, ranging from southern Maine south along the Appalachian Mountain chain to northern Georgia and west into Ohio ( USDA Forest Service 2011 ). Throughout much of this range, it has caused widespread decline and mortality among populations of eastern hemlock *(T. canadensis* (L.) Carr.) and Carolina hemlock *(T. caroliniana* Engelm.).

In the eastern United States, *A. tsugae* has a complex polymorphic life cycle that includes two wingless parthenogenetic generations and one winged sexual generation each year ( McClure 1989 ). The first appearance of each generation and the timing of life stages varies considerably with latitude ( McClure 1989 ; Gray and Salom 1996 ; Joseph et al. 2011 ). The two parthenogenic generations, called the progrediens and sistens, are present from March to June and June to March, respectively. The winged sexual generation is called the sexuparae and overlaps with the progrediens. Upon molting to the adult form, the sexuparae migrate away from hemlock for sexual reproduction on spruce *(Piceae* spp.); however, the native spruce flora of eastern North America have thus far been immune to *A. tsugae* infestation and successful sexual reproduction has never been observed in the introduced range.

The first instar nymphs of the progrediens and sistens generations are called crawlers and are the only mobile stage of the insect. After hatching, crawlers move about their natal trees or passively disperse to nearby trees in search of feeding sites on the pulvinus (needle cushion) at the base of hemlock needles, where the insect remains for the duration of its life ( Oten 2011 ). Adelgids feed by inserting their stylets (piercing/sucking mouthparts) into the plant and extracting stored nutrients from xylem ray parenchyma cells ( Young et al. 1995 ). Feeding causes changes in hemlock cellular structures and water potential ( Walker-Lane 2009 ; Gonda-King et al. 2012 ) and leads to the abortion of reproductive and vegetative buds, restriction of new growth, dessication, and needle loss. Severe infestations can kill trees in as few as four years, although some trees have been noted to survive infestation for more than 10 years ( McClure et al. 2001 ).

The integrated strategy to manage the impact of *A. tsugae* on eastern forests includes classical biological control, chemical insecticides, silvicultural practices, gene conservation, and host resistance ( Onken and Kenna 2008 ). Of these, biological and chemical control have received the most interest; biological control currently is considered the most promising tool for long-term adelgid management ( Onken and Reardon 2011 ). Since 2002, however, the general understanding of host-insect interactions in the hemlock-adelgid system has improved, and the possibility exists for finding or breeding *A. tsugae-resistant* hemlock genotypes for reforestation. Initially, both hemlock species were thought to be universally susceptible to *A. tsugae* attack ( McClure 1992 ), but recent evidence suggests that some level of endogenous adelgid tolerance or resistance may exist within Eastern hemlock ( Caswell et al. 2008 ) and Carolina hemlock ( Jetton et al. 2008 , Oten 2011 ). Attempts to breed the adelgid resistance of *T. chinensis* (one of the Asian hemlocks) into Carolina hemlock through interspecific hybridization were also successful ( Montgomery et al. 2009 ).

The screening of tree genotypes for pest resistance is often accomplished with young plants derived from seed or clonally propagated via rooted stem cuttings or grafted scions. Upon reaching suitable age or size, these plants are artificially infested or inoculated with the insect or pathogen of concern. Examples include the selection of pine *(Pinus* spp.) genotypes for resistance to pitch canker *(Fusarium circinatum)* and fusiform rust *(Cronartium quercuum),* and poplar *(Populus* spp.) hybrids for resistance to the cottonwood leaf beetle, *Chrysomela scripta* F. ( Foster and Anderson 1989 ; Bingaman and Hart 1992 ; Hodge and Dvorak 2007 ). Within the context of an operational tree breeding program, a key to the success of insect resistance screening is that large numbers of genotypes can be tested in a time- and cost-efficient manner. It is also important that infestation rates are consistent across plants and population pressure is sufficient to induce plant responses ( Smith et al. 1994 ). Within the hemlock- *A tsugae* system, direct infestation of individual seedlings or clones with standard numbers of insects has been evaluated experimentally ( Butin et al. 2007 ) and used successfully to select for variation in adelgid resistance within and among hemlock species ( Caswell et al. 2008 ; Jetton et al. 2008 ). Although successful in these small studies, the time necessary for counting and placing adelgids on individual plants is unlikely to meet the time and cost efficiency needs of larger breeding programs that seek to screen among thousands of hemlock genotypes simultaneously.

The development of artificial infestation techniques that are efficient and reliable for screening large numbers of putatively adelgid resistant genotypes has been identified as a research priority by the USDA Forest Service Working Group on Genetics and Host Resistance in Hemlock. To address this priority, the preliminary experiments reported here were designed to test the utility of a rain-down technique for inducing artificial adelgid infestations on hemlock seedlings en masse. The obj ective of the study was to evaluate the density and distribution patterns of *A. tsugae* progrediens and sistens crawlers allowed to rain down onto glue sheets and hemlock seedlings.

## Materials and Methods

### *A. tsugae* source material

This study consisted of two separate infestation experiments in the spring of 2011, one using *A. tsugae* crawlers of the progrediens generation (March), and the other using crawlers of the sistens generation (May). For the March experiment, *A. tsugae* ovisacs containing sistens adults and progrediens eggs were collected on 18 March 2011 from eastern hemlocks along Bletchers Creek near Petros, TN (36.09395°N, -84.409075°W). Pole prun-ers were used to cut infested branches (0.6-1.2 m long) from hemlocks that were not yet in visible stages of decline. Smaller branch tips (40 cm long) were cut from this material, and the cut ends were placed in 19 L buckets filled 3 cm deep with cold tap water. Branch tips were kept in water indoors under air conditioning (18.3°C) until used in the experiment. Branches infested with progrediens adults and sistens eggs were collected on 18 May 2011 from the same site as the March experiment and were processed as described above. For each experiment, density of *A. tsugae* on the source material was estimated by counting the total number of ovisacs on each of 15 branch tips, and the number of eggs per ovisac was estimated by sub-sampling 10 ovisacs on each branch ( Table 1 ).

**Table 1.**
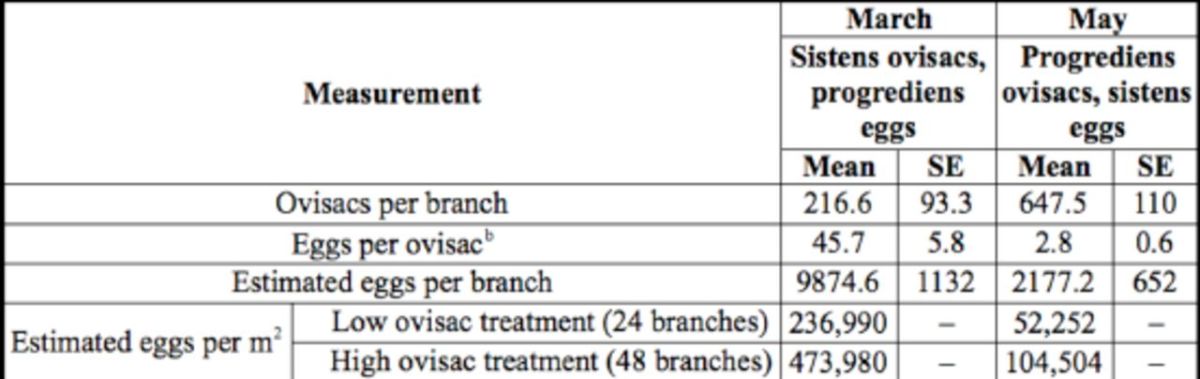
Mean (SE) hemlock woolly adelgid (HWA) ovisac and egg densities on 40 cm-long eastern hemlock branches ^a^ used during infestations experiments in March and May 2011.

^a^ In both the March and May experiments, ovisacs were counted on 15 40-cm branch samples collected from infested hemlocks near Petros, TN.

^b^ Eggs were counted within 10 ovisacs on each of 15 branches.

### Progrediens rain down experiment (March)

Experiments were conducted at the North Carolina Department of Agriculture and Consumer Services/North Carolina State University Mountain Research Station in Waynesville, NC (35.487319°N, ‒82.966883°W). In the March experiment, 10 cubic cages (1 × 1 × 1 m) built of 2-cm diam schedule-40 PVC pipe were placed in a non-climate-controlled building subject to outdoor temperatures but shielded from wind and precipitation. Cages were covered on the top with poultry wire and on the four vertical sides with clear 6 mil plastic sheeting. The cage was placed on concrete floor over a 1 m ^2^ sheet of paper printed with a 10 × 10 cm grid (100 squares). Sixty percent of the sheet (60 × 100 cm) was covered with a thin layer of insect glue (Tanglefoot, Contech Enterprises, Victoria, BC) to catch crawlers; the remaining portion (40 × 100 cm) was used for seedling placement. On 22 March 2011, the 40-cm infested eastern hemlock branch tips were placed bottom side down on the poultry wire on the top of each cage. Cages were arranged in five rows of two paired cages. One cage in each pair received a “low” ovisac treatment of 24 branch tips (evenly distributed in three rows of eight branches), and the other received a “high” ovisac treatment of 48 branch tips (three rows of 16 branches). The low and high ovisac treatments were estimated to represent placement of ≈237,500 and 475,000 eggs, respectively, on each cage ( Table 1 ). The position of the high- and low-treatment cages within each pair was alternated systematically from row to row. Coincident with branch tip placement, one Carolina hemlock seedling (1.65 L pot) was placed beneath each cage, and one eastern hemlock seedling (1.65 L pot) was placed next to each Carolina hemlock seedling nine days later (the eastern hemlock seedlings were not available at the beginning of the experiment).

After 21 days (12 April 2011), seedlings and the gridded glue sheets were removed from the cages. Although cages were shielded from direct precipitation, one pair of glue sheets was discarded because of damage caused by water leaking from a nearby window onto the floor during a severe storm. The density of progrediens crawlers raining down was estimated by using a subsampling scheme. The 60 × 100 cm glue sheet was divided into 15 20 × 20 cm sheets, and each of these sheets was further subdivided into 100 2 × 2 cm squares. Four 2 × 2 cm squares were randomly selected from each of the 20 × 20 cm sheets, and the number of crawlers in each square was counted under a dissecting microscope. All Carolina and eastern hemlock seedlings were placed under a covered outdoor porch and watered weekly until early June, when they were sampled for *A. tsugae* density. Three seedlings (two Carolina hemlocks and one eastern hemlock) died before sampling and were excluded from analysis. Nine 10-cm long branch tips were clipped from each surviving seedling, one from the terminal shoot and four from lateral shoots in the upper and lower halves of the seedling. Lateral shoot samples were separated by ≈90°. Each 10 cm branch tip was examined under a dissecting microscope, and the number of settled progrediens adults was counted.

### Sistens rain down experiment (May)

The May experiment was conducted at the same location as the March experiment using 12 PVC cages identical to those described above. Eight of the cages were arranged in four rows of two paired cages, with one cage in each pair receiving the low (24 branches) ovisac treatment and the other receiving the high (48 branches) ovisac treatment, as described previously. These cages were used to assess density of sistens crawlers raining down onto a 1 m2 sheet of paper printed with a 10 × 10 cm grid (100 squares). In this experiment, the entire 1 m2 sheet was coated with insect glue to assess crawler density. The remaining four cages (arranged as two blocks of two cages) were used to infest Carolina hemlock seedlings in 1.65 L pots (two seedlings beneath each cage). On 24 May 2011, 40 cm eastern hemlock branch tips infested with progrediens adults and sistens eggs were placed on the cages in the same manner as described for the March experiment.

After 10 days (2 June 2011), seedlings and the gridded glue sheets were removed from the cages. Each 1 m2 glue sheet was divided into 25 20 × 20 cm sheets, and each of these sheets was further subdivided into 100 2 × 2 cm squares. Twenty 2 × 2 cm squares were randomly selected from each of the 20 × 20 cm sheets, and the number of crawlers in each square was counted under a dissecting microscope. Carolina hemlock seedlings were placed under a covered outdoor porch and watered weekly until mid August 2011, when they were sampled for settled *A. tsugae* sistens nymph density using the same method described for the March experiment. The estimated numbers of ovisacs and eggs applied to each treatment are listed in Table 1 .

### Data analysis

Mean densities of adelgid crawlers (on glue sheets) and settled nymphs and adults (on seedlings) were calculated for each treatment. For the March and May experiments, a paired *t* -test for dependent samples was used to test the null hypothesis that ovisac treatment level had no effect on mean crawler density on gridded glue sheets. The spatial distribution of crawlers was analyzed visually by computing mean crawler density on each 20 × 20 cm section of the glue sheet and graphically shading blocks of the 1 m2 grid according to relative density categories. When we compared settled progrediens adult density on seedlings in the March experiment, seedling mortality prevented use of a paired *t* -test, and thus ovisac treatment means were compared using a *t* -test for independent samples. In the March experiment, mean adelgid densities were not compared between hemlock species (Carolina and eastern) because the eastern hemlock seedlings were exposed to the treatments for a shorter period of time. Analysis of variance (ANOVA) was used to test the effect of block and ovisac treatment, their interaction, and the effect of seedling nested in the block by ovisac treatment interaction on the density of settled sistens nymphs on Carolina hemlock seedlings in the May experiment. Data were analyzed using Statistica v. 9.1 (Statsoft, Tulsa, OK).

## Results

### Progrediens rain down experiment (March)

**Figure 1.**
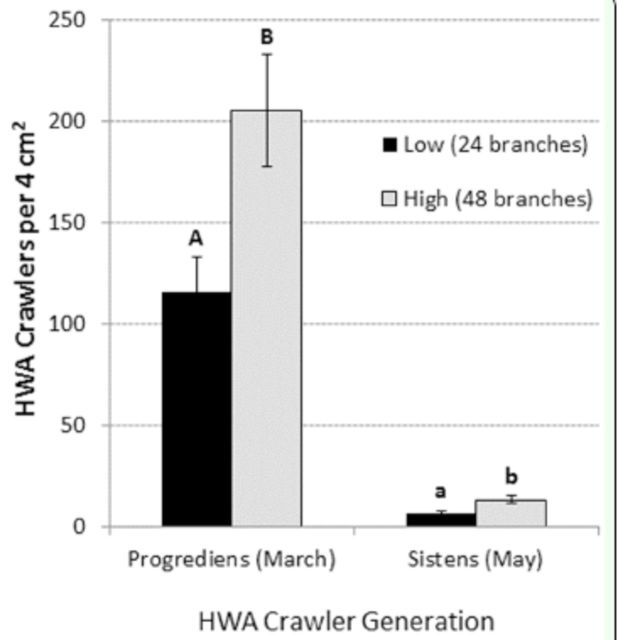
Mean (SE) number of HWA crawlers per 2 * 2 cm sample that rained down onto glue sheets for each adelgid generation (progrediens and sistens) and each ovisac density treatment (low and high). Bars with different letters of the same **I** case are significantly different at **a ^** 0.05.I

**Fig 2.**
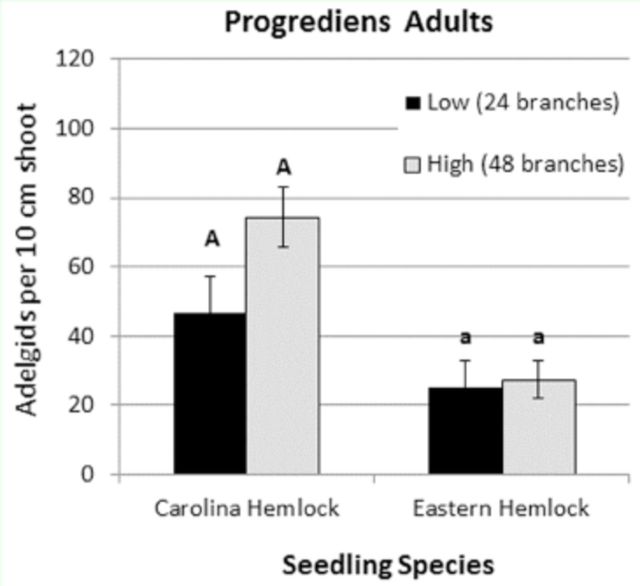
Mean (SE) density of hemlock woolly adelgid progrediens adults on Carolina and eastern hemlock seedlings exposed to progrediens crawlers in the March 2011 rain down experiment. Bars with different letters of the same case are significantly different at α ≤ 0.05.

The high ovisac density treatment (48 infested branches) resulted in significantly more progrediens crawlers raining down per unit area than the low ovisac density treatment (24 infested branches) *(t* = —5.32; df = 3; *P* = 0.013) ( Fig. 1 ). There was no significant effect of ovisac density treatment on mean adult progrediens density settled on Carolina hemlock *(t* = 1.99; df = 6; *P* = 0.094) or eastern hemlock *(t* = 0.26; df = 7; *P* = 0.805) seedlings. Mean density of progrediens adults on Carolina hemlock seedlings ranged from 47 to 74 adelgids per 10 cm shoot beneath the low and high ovisac density treatments, respectively ( Fig. 2 ). Mean progrediens densities on eastern hemlock seedlings, which were exposed to the treatments for nine fewer days than the Carolina hemlock seedlings, were about 25 adelgids per 10 cm ( Fig. 2 ).

### Sistens rain down experiment (May)

**Figure 3.**
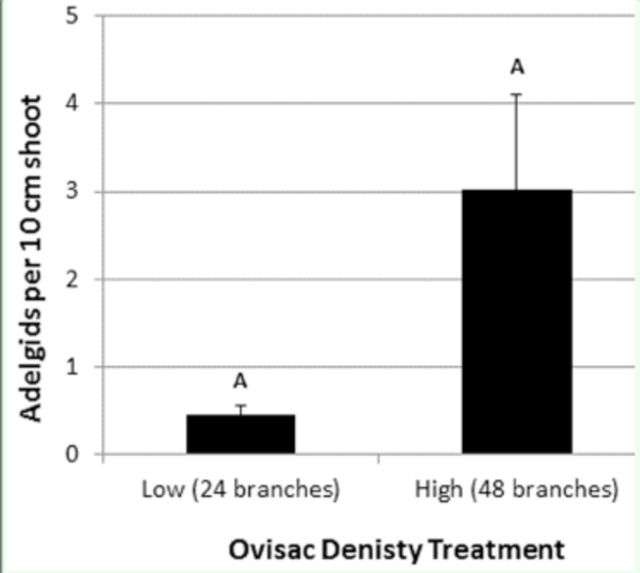
Mean (SE) hemlock woolly adelgid sistens nymphs settled on Carolina hemlock seedlings exposed to sistens crawlers in the May 2011 rain down experiment. Bars with different letters are significantly different at **a ^** 0.05.

The high ovisac density treatment (48 infested branches) resulted in significantly more sistens crawlers raining down per unit area than the low ovisac density treatment (24 infested branches) *(t* = —3.28; df = 3; *P* = 0.046) ( Fig. 1 ). Sistens crawler density in the May experiment, however, was more than an order of magnitude lower than progrediens crawler density in the March experiment ( Fig. 1 ). The density of sistens nymphs settled on Carolina hemlock seedlings did not differ significantly with block (F = 0.41; df = 1,4; *P* = 0.558), ovisac treatment (F = 2.87; df = 1,4; *P* = 0.165), the block by ovisac treatment interaction (F = 0.61; df = 1,4; *P* = 0.477), or seedling nested in the block by ovisac treatment interaction (F = 2.17; df = 4,64; *P* = 0.083). Densities of settled sistens were extremely low, averaging only three adelgids per 10 cm shoot in the high ovisac treatment ( Fig. 3 ), roughly an order of magnitude lower than settled progrediens densities in the March experiment ( Fig. 2 ).

## Discussion

The results of this study suggest that the crawler rain down technique is suitable for adequately infesting hemlock seedlings with *A. tsugae* . In the March experiment, after evenly covering the poultry-wire-topped PVC cages with *A. tsugae-* infested branch tips, first instar adelgid nymphs (crawlers) of the progrediens generation rained down and were well-distributed over the 1 m ^2^ gridded glue surfaces placed below the cages. As expected based on ovisac and egg abundance counts on the *A. tsugae-* infested source material ( Table 1 ), crawler density on the grids in the high ovisac density treatment (205.59/4 cm ^2^ ) was nearly twice that in the low ovisac treatment (115.98/4 cm ^2^ ). Extrapolating from these sub-sampling means, it is estimated that 289,000 (low treatment) to 513,000 (high treatment) progrediens crawlers rained down per m ^2^ . These crawler densities are at the high end of the range of 36 to 205/4 cm ^2^ reported in a similar study where crawlers of the balsam woolly adelgid, *Adelges piceae* (Ratzeburg), were allowed to rain down onto a gridded glue surface from an adelgid infested log of Fraser fir, *Abies fraseri* (Pursh) Poir., suspended above ( Newton et al. 2011 ).

The rain down of *A. tsugae* progrediens crawlers resulted in the successful infestation of the eastern and Carolina hemlock seedlings placed in each cage. Seedling infestation rates for Carolina hemlock ranged from 46.8/10 cm in the low ovisac treatment to 74.3/10 cm in the high ovisac treatment, at or above the four adelgids per cm threshold necessary for inhibiting new growth and causing visible signs of decline in hemlock ( McClure 1991 ). This result is encouraging for the utility of the rain down technique to infest hemlocks for adelgid resistance screening. However, because each cage contained only two seedlings, it is unclear whether this approach to artificial infestation will produce consistent infestation density and sufficient population pressure when a larger number of seedlings are placed in each cage, as would be necessary in a large scale resistance breeding program.

Interestingly, although *A. tsugae* infestation rates on Carolina hemlock were 1.5 times higher in the high ovisac density treatment compared with the low ovisac treatment, this difference was not statistically significant. This lack of statistical difference may be attributable to the small number of seedlings evaluated, but it may also suggest that the lower density 24 branch treatment might be sufficient for inducing adelgid infestations at damage threshold levels when using the rain down approach. Similar findings have been reported in studies testing the direct infestation of individual plants with *A. tsugae* , where resulting infestation densities did not differ between treatments of 50 versus 100 ovisacs placed on eastern, Carolina, and western hemlock seedlings in a greenhouse ( Jetton et al. 2008 ), or when one versus three 20 cm *A. tsugae-* infested hemlock branch tips were placed on mature eastern hemlocks growing in a Massachusetts forest ( Butin et al. 2007 ). However, the efficiency of the low versus high ovisac density treatments in the rain down approach also needs additional testing in cages containing larger numbers of seedlings.

Infestation rates on eastern hemlock seedlings in the March experiment were below McClure’s (1991) damage threshold, and, similar to Carolina hemlock, did not differ significantly between the high and low ovisac treatments. The best explanation for the lower infestation rates recorded on eastern hemlock is that, because of availability, eastern hemlock seedlings were not placed in cages until nine days after the start of the experiment, meaning they were exposed to adelgid crawlers for only 12 days of the 21-day experiment and likely missed the period of peak crawler emergence and settlement. In other studies that used cut hemlock branch tips with mature oviacs as the infestation source, crawler settlement rates tended to be highest during the first 16 days after cutting ( Butin et al. 2007 ), and crawler emergence peaked on the fifth day after cutting (ZLP., unpublished data).

Hemlock woolly adelgid progrediens adults that give rise to sistens crawlers in May have lower fecundity than sistens adults that give rise to progrediens crawlers in March ( McClure 1989 ). Thus, it is sistens ovisacs available in early spring that are typically recommended for direct infestations on individual hemlocks to ensure adequate population pressure ( Butin et al. 2007 ). Based on the results of the May experiment in this study, the same recommendation applies for seedling infestations via the rain down method. Sistens crawler densities on grids below the low and high ovisac treatments were, on average, 16 times lower compared with progrediens crawler densities in the March experiment. The overall lower abundance of sistens crawlers raining down in the May experiment also resulted in substantially lower infestation rates on the Carolina hemlock seedlings that were well below the *A. tsugae* damage threshold ( McClure 1991 ).

The 16-fold reduction in crawler density between the March and May experiments ( Fig. 1 ) corresponded with a 16-fold reduction in egg density on the hemlock source material between the two experiments ( Table 1 ). This fecundity differential between the two *A. tsugae* generations is much greater than reported by McClure (1989) , who recorded 48.6 and 21.7 eggs per ovisac for sistens and progrediens adults, respectively, which suggests that additional factors may have negatively influenced the reproductive success of adelgids used in our May experiment. One possibility is that intense competition for food resources affected the fecundity of the progrediens adults, which were at three times the mean density on the May source material compared to the density of sistens adults on the March material ( Table 1 ). Also, high densities of sexuparae are indicative of declining health in source trees and related reductions in fecundity in *A. tsugae* infestations ( McClure 1991 ). The source material used in the May experiment produced an abundance of the sexuparae generation, as evidenced by a substantial density of alates (3.68 sexpaurae per 4 cm ^2^ averaged across treatments) falling onto the glue sheets along with the sistens crawlers in May. The use of healthier host material and/or host material with a lower density of progrediens adults may have resulted in better adult fecundity and higher sistens crawler densities.

**Figure 4.**
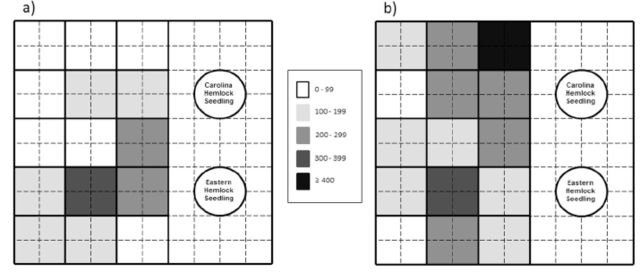
The spatial distribution (mean density on each 20 × 20 cm section ) of progrediens crawlers on glue sheets in the March 201 1 rain down experiment for the low (a) and high (b) ovisac density treatments.

**Figure 5.**
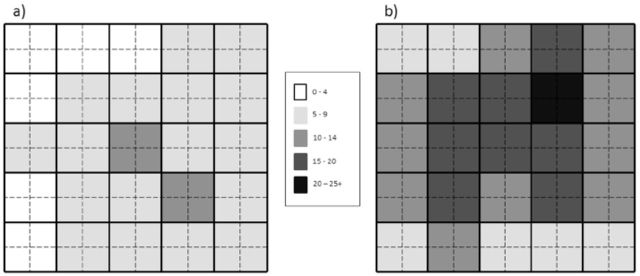
The spatial distribution (mean density on each 20 × 20 cm section) of sistens crawlers on glue sheets in the May 2011 rain down experiment for the low (a) and high (b) ovisac density treatments.

Newton et al. (2011) found that when a Fraser fir log infested with balsam woolly adelgid was suspended over a gridded glue sheet, falling crawlers were concentrated in a 30 cm wide band directly below the log, with very light crawler densities toward the edges. This is problematic from the stand point of host resistance screening because seedlings placed directly below the log are likely to become more heavily infested than those placed toward the edges, resulting in uneven population pressure and confounded determinations of resistance or susceptibility. One hypothesis for this study was that, because infested hemlock branches were distributed evenly over the m ^2^ grids, *A. tsugae* crawler distribution and abundance would be more homogeneous. As seen in Figures 4 and 5 , this was not the case. Even with a systematic and even distribution of branches, “hot spots” of unusually high crawler density occurred in both ovisac density treatments in the March and May experiments, and higher crawler densities were frequently (but not always) found toward the center of the sheet as opposed to near the edges. This aspect of the rain down technique needs to be refined to promote a more homogeneous distribution of crawlers within infestation cages before this method can be deployed in an *A. tsugae* resistance screening and breeding program for eastern and Carolina hemlock.

## Conclusions

These preliminary studies on the development of a rain down technique for artificially infesting eastern and Carolina hemlock seedlings with *A. tsugae* are encouraging and suggest that this might be a suitable approach for mass inoculations for resistance screening. In both experiments, *A. tsugae* crawlers emerged and dispersed from adelgid-infested branch tips placed on top of the PVC cages and fell in large numbers into the space below. The data indicate that progrediens crawlers emerging in March rained down at substantially higher densities and resulted in higher *A. tsugae* infestation rates on seedlings compared with sistens crawlers emerging in May. Although crawlers were well-distributed across the gridded glue sheets in March and May, hot spots of unusually high density that could bias results of resistance screening occurred in both experiments. Additional studies to test the utility of this approach for mass artificial infestations are ongoing at the NCDAͨ/NCSU Mountain Research Station. The experiments are evaluating the ability of the rain down technique to induce *A. tsugae* infestations of consistent population density and pressure on multiple hemlock seedlings of three species (eastern, western, and Carolina), and are testing moving versus stationary cages for promoting more homogeneous crawler distributions.
